# Organ microenvironment affects growth and metastasis of hepatocellular carcinoma via the TGF-β/Smad pathway in mice

**DOI:** 10.3892/etm.2012.752

**Published:** 2012-10-18

**Authors:** GUOCAI LI, LUNXIU QIN, QINGHAI YE, QIONGZHU DONG, NING REN, HULIANG JIA

**Affiliations:** 1 Liver Cancer Institute, Zhongshan Hospital, Fudan University, Shanghai 200032;; 2 Gaoxin Hospital, Xi’an Jiaotong University, Xi’an, Shanxi, P.R. China

**Keywords:** hepatocellular carcinoma, metastasis, transforming growth factor-β, organ microenvironment

## Abstract

The interaction between cancer and the organ microenvironment is complex, and the transforming growth factor-β (TGF-β)/Smad pathway plays an important role in this interaction. However, the role of the organ microenvironment in hepatocellular carcinoma (HCC) is not well understood. To evaluate the effect of the organ microenvironment and the role of the TGF-β/Smad pathway, MHCC97-H cells were inoculated subcutaneously into nude mice and the resulting MHCC97-H subcutaneous tumor tissues were implanted into the livers of the mice. We found a higher tumor weight and less pulmonary metastasis for the cancers in liver sites than for those in subcutaneous sites; the TGF-β1 levels were significantly different between the tumor models and correlated with tumor metastasis. Our results suggest that the organ microenvironment affects the growth and invasion of liver cancer cells. The TGF-β/Smad pathway is significant in the interaction between HCC and its microenvironment and affects the progression of HCC.

## Introduction

Hepatocellular carcinoma (HCC) is one of the most common types of cancer in the world and a significant cause of mortality in sub-Saharan Africa and Eastern Asia (1). The overall five-year survival rate following resection has remained as low as 35–50% (2–4). The extremely poor prognosis of HCC is largely the result of a high rate of recurrence following surgery and of metastasis (5,6). Exploring the mechanisms involved in the process of HCC metastasis is vital as it may provide new therapeutic targets for HCC and is likely to be useful in further improving the long-term survival of patients with HCC (7).

It is well documented that the specific organ microenvironment not only affects the proliferation, angiogenesis and invasion of cancer but also influences the expression of metastasis-regulating genes (8,9). Malignant cells in solid tumors communicate with the microenvironment via a complex network of extracellular signals, which includes a large number of cytokines (10). Therefore, investigation of the role of these cytokines in the interaction between the organ microenvironment and the tumor may aid the revelation of the mechanism of tumor metastasis. Transforming growth factor-β (TGF-β) is a known regulator of epithelial cells and of autonomous tumor initiation, progression and metastasis (11–13). Components of the TGF-β/Smad pathway are considered to be major tumor suppressor genes; the absence or malfunction of the genes is believed to lead to loss of growth regulation. Previous studies have indicated that TGF-β is significantly involved in the interactions between cancer cells and the tumor microenvironment. Specifically, the loss of TGF-β signaling in stromal components may result in an ‘activated’ microenvironment that supports and initiates the transformation of adjacent epithelial cells (14). In HCC, TGF-β is a useful serological marker for the early detection of cancer (15) and plays a dual role in the progression of HCC. TGF-β is able to stimulate non-invasive HCC cells to acquire invasive phenotypes (16) and also induces *in vitro* apoptosis of hepatoma cells (17).

However, little is known regarding the interaction between the organ environment and HCC. Moreover, the role of TGF-β/Smad in the course of HCC has yet to be elucidated. In the current study, we demonstrate that the organ microenvironment regulates the growth and invasion of liver cancer cells via TGF-β/Smad.

## Materials and methods

### Cell lines and culture

The MHCC97-H cell line was established from human HCC cells in the Live Cancer Institute of Fudan University (Shanghai, China) (18,19). The cells were cultured in high glucose Dulbecco’s modified Eagle’s medium (H-DMEM; Gibco-BRL, Carlsbad, CA, USA) and supplemented with 10% fetal calf serum (Gibco-BRL) at 37°C in a humidified incubator containing 5% CO_2_.

### Establishment of animal models of HCC

BALB/c nude mice, average weight 25 g, were used in this experiment. MHCC97-H models were established by inoculating 6×10^6^ MHCC97-H cells subcutaneously into the right sides of the backs of the nude mice (n=26). Xenograft models were established (n=17) via orthotopic implantation of MHCC97-H subcutaneous tumor tissues (volume ∼2×2×1 mm^3^) into the livers of the mice as previously reported (20). These experiments were approved by the Shanghai Medical Experimental Animal Care Commission.

### Collection of samples and analysis of pulmonary metastasis

After feeding for 35 days, the animals were sacrificed. The tumor tissues were removed and weighed. The lungs were removed, fixed in paraformalin and embedded in paraffin. Each sample was sliced into 20 sections, each 5 *μ*m in thickness with 50-*μ*m intervals between successive sections. After staining with hematoxylin and eosin (H&E), the sections were independently observed under a microscope by two pathologists to evaluate pulmonary metastasis.

### RNA extraction and real-time PCR

The total RNA of the tumor tissues was extracted using the TRIzol reagent (Invitrogen Life Technologies, Carlsbad, CA, USA) according to the instructions of the product. Using SYBR-Green mix (Toyobo Co., Ltd., Osaka, Japan), real-time RT-PCR analysis was performed to identify the expression levels of TGF-β, Smad2 and Smad7. The primers were designed by software (Primer Premier 5.0) as follow: TGF-β sense, 5′-GGC GATACCTCAGCAACCG-3′ and antisense, 5′-CTAAGG CGAAAGCCCTCAAT-3′; Smad2 sense, 5′-TACTACTCT TTCCCCTGT-3′ and antisense, 5′-TTCTTGTCATTTCTA CCG-3′; Smad7 sense, 5′-CAACCGCAGCAGTTACCC-3′ and antisense, 5′-CGAAAGCCTTGATGGAGA-3′; and β-actin sense, 5′-TCGTGCGTGACATTAAGGAG-3′ and antisense, 5′-ATGCCAGGGTACATGGTAAT-3′. The amplification conditions were: 95°C for 9 min, followed by 45 cycles of 95°C for 30 sec, 57°C for 30 sec and 72°C for 15 sec, followed by an extension at 72°C for 5 min. β-actin was used as a control for the presence of amplifiable cDNA. The mRNA expression level was assessed by 2^−ΔΔCt^. In brief, the Ct value for the target gene was subtracted from the Ct value of β-actin to yield a ΔCt value. The average ΔCt was calculated for the control group and this value was subtracted from the ΔCt of all other samples (including the control group). This resulted in a ΔΔCt value for all samples which was then used to calculate the fold-induction of mRNA expression of the target gene using the formula 2^−ΔΔCt^, as recommended by the manufacturer (Bio-Rad, Hercules, CA, USA). In the current study, MHCC97-H model samples were used as the control.

### Protein extraction and western blot analysis

Sections of frozen tumor samples (n=14) were lysed in RIPA buffer (50 mM Tris-HCl pH 7.5; 150 mM NaCl; 0.5% NaDOC; 1% NP-40; and 0.1% SDS) with protease inhibitors. Protein was extracted by spinning in a microcentrifuge for 30 min. Protein concentrations were determined using the Bradford reagent. Equal amounts of each sample (20 *μ*l) and 10 *μ*l markers were run on 10% SDS-PAGE gels and electro-transferred onto PVDF membranes using the Mini-Genie blotting system (Bio-Rad). The membranes were incubated with primary antibody, mouse anti-human TGF-β1 antibody (Chemicon, Temecula, CA, USA; 1:1000 diluted), mouse anti-human β-actin antibody (Chemicon; 1:2000 dilution) and HRP-conjugated goat anti-mouse IgG secondary antibody (Sigma, St. Louis, MO, USA; 1:2000 dilution), The membranes were washed, incubated with 10 ml LumiGLO and exposed to film.

### Immunohistochemistry

Paraffin-embedded tumor tissues were sliced into 5 *μ*m-thick sections and mounted on glass. The slides were deparaffinized and rehydrated over 10 min through a graded alcohol series to deionized water; 1% Antigen Unmasking solution (Vector Laboratories, Burlingame, CA, USA) and microwave treatment were used to enhance antigen retrieval. The slides were incubated with the specific TGF-β1 primary antibody and with HRP-conjugated secondary antibody and then stained with DAB.

### ELISA

The total protein of all tumor tissues was extracted as described in a previous section. TGF-β1 protein levels in the tumors were determined using the Quantikine ELISA TGF-β1 immunoassay kit (R&D Systems, Minneapolis, MN, USA). The operational approach was according to the manufacturer’s instructions.

### Statistical analysis

Statistical analysis of the data was performed using SPSS 11.5 software (SPSS, Inc., Chicago, IL, USA). The correlation between TGF-β/Smad and tumor weight was estimated using linear regression analysis and correlation coefficients were evaluated by the Student’s t-test. Multiple covariance analysis was used for interaction between TGF-β/Smad and metastasis. All statistical tests were two-sided; P<0.05 was considered to indicate a statistically significant result.

## Results

### Tumor weight and pulmonary metastasis in the animal models

The mean tumor weights (g) in the MHCC9-H and xenograft models were 1.83±0.75 and 2.89±0.84, respectively (P<0.01). The pulmonary metastatic rate and the number of metastatic foci in the MHCC97-H model were higher than those in the xenograft model (69.2 vs. 64.7%; and 4.72±5.50 vs. 2.27±1.01, respectively) but the difference was not statistically significant, while the number of metastatic cells approximated a statistically significant difference (119.11±185.92 vs. 85.18±79.96, respectively, P=0.08; Table I).

### Organ microenvironment may affect the expression of TGF-β1 and Smad

**Figure 1:**
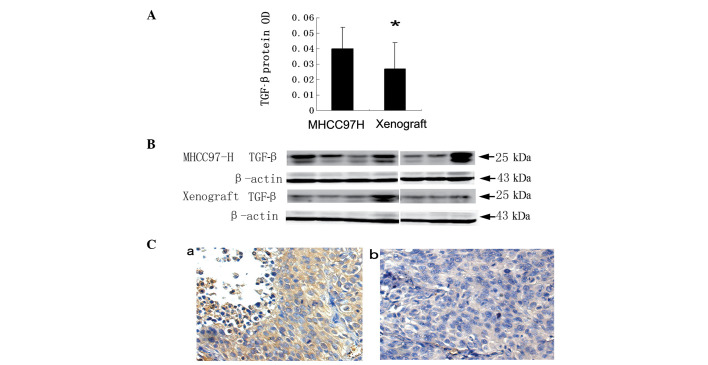
TGF-β protein levels in two animal models. (A) TGF-β protein levels in the MHCC97-H model were higher than in the xenograft model by ELISA, ^*^P<0.05. (B) TGF-β protein levels in the MHCC97-H model were higher than in the xenograft model by western blot analysis. (C) The expression of TGF-β1 in (a) the MHCC97-H model and (b) the xenograft model by immunohistochemical staining. The brown-yellow color is positive staining (x20 objective field). TGF-β1, transforming growth factor-β1.

After implanting the MHCC97-H subcutaneous tumor into the mouse liver, the mRNA levels of TGF-β1 were significantly decreased (1.92±1.70 vs. 0.97±0.73; P=0.035). However, the Smad2 and Smad7 mRNA levels in the MHCC97-H model were not statistically different from those in the xenograft model (Table II). The protein levels of TGF-β1 in the MHCC97-H model mice were revealed to be higher than those in the xenograft model mice by ELISA with OD values of 0.04±0.01 and 0.03±0.01, respectively, (P=0.003; Fig. 1A); similar results were obtained by western blotting (Fig. 1B) and immunohistochemical staining (Fig. 1C).

### Expression of Smad7 correlated with tumor size

**Figure 2:**
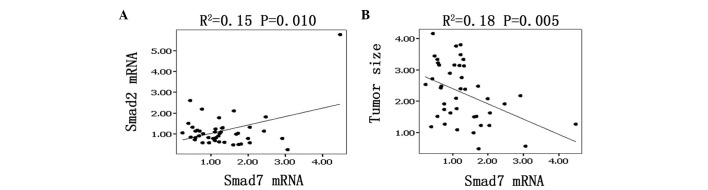
Correlation between Smad and tumor weight. (A) The expression of Smad2 and Smad7 was linearly correlated by regression analysis. (B) Smad7 expression was linearly correlated with tumor weight. Dots denote the samples. The lines are regression lines. R, correlation coefficient; P, P-value.

The mRNA levels of Smad7 linearly and positively correlated with those of Smad2 by linear regression analysis. The correlation coefficient R^2^=0.15, which has a statistical significance according to the Student’s t-test (P=0.005; Fig. 2A). Moreover, Smad7 mRNA levels were linearly and negatively correlated with tumor weight (R^2^=0.18, P=0.005; Fig. 2B).

### Expression of TGF-β1 mRNA correlated with metastasis

We divided all the samples (n=43) into three groups according to the median metastatic cell number: non-metastatic, lower metastatic and higher metastatic groups. We identified that the higher metastatic group had a higher mean TGF-β level than the non-metastatic and lower metastatic groups by multiple covariance analysis. The mean TGF-β1 mRNA levels were 2.32±2.11, 1.10±0.83 and 1.16±0.63, respectively, P= 0.024 (Table III).

## Discussion

Paget’s ‘seed and soil’ hypothesis suggests that the interaction between tumor cells and target organ determines whether metastasis will occur (21). Metastasis depends on multiple interactions of cancer cells with host homeostatic mechanisms. In this present study, when the subcutaneous tumor tissues were transplanted into the liver, pulmonary metastasis was reduced. These results suggest that the organ microenvironment may alter the invasive potential of HCC.

We also found that the expression levels of TGF-β1 in the MHCC97-H and xenograft models were statistically different. In addition, TGF-β1 was highly expressed in the higher metastatic group and the expression of Smad7 negatively correlated with tumor size. These result indicate that the TGF-β/Smad pathway plays an important role in the interaction between HCC and the organ microenvironment and affects the progression of HCC. Similar results have been published for renal cancer, which demonstrate that the basic fibroblast growth factor (bFGF) levels of tumors implanted in kidney were 10–20 times higher than in subcutaneous tumors (22). Other studies have revealed that tumors growing in the stomach express more vascular endothelial cell growth factor (VEGF) than ectopically placed tumors and only the tumors in the stomach were able to undergo metastasis (6,23).

It has been reported TGF-β plays a dual role in the progression of tumors. During the early stages of tumor formation, TGF-β acts as a tumor suppressor, inhibiting proliferation and inducing apoptosis of tumor cells. However, during the later stages of tumorigenesis, a number of tumor cells become unresponsive to the growth inhibitory functions of TGF-β and become more motile and invasive (24). Our findings that the location of the tumor in the liver correlated with bigger size and lower metastasis are consistent with the dual role of TGF-β1 following implantation and support this view.

The molecular mechanism for the downregulation of TGF-β1 production in HCC in the liver remains unclear. With the exception of the delicate balance between TGF-β and the tumor microenvironment (14), TGF-β1 expression is affected by various growth factors secreted by normal and tumor cells. Signaling by TGF-β family members occurs mainly through Smad proteins (25), and TGF-β and Smad may cross-talk with other pathways (26,27). Future studies are necessary to determine whether the expression levels of other factors are also modulated by the organ microenvironment.

A number of tumors have a selectivity for metastasis to specific organs; the precise cellular and molecular mechanisms involved are unknown. It has been reported that differences in tumor-secreted humoral factors, the upregulation of fibronectin and site-specific delivery of VEGFR1^+^ cells within target organs may promote metastatic spread in specific distant organs (28). Our results indicate that TGF-β1 is significant in the preference for metastasis to the lung.

The results of the current study suggest that the organ environment affects the progression of HCC. For many years, all efforts to treat cancer have concentrated on the inhibition or destruction of tumor cells, but none of them have been able to alter the natural history of the disease (8,29). Strategies to modulate the TGF-β levels of the host microenvironment may provide a better approach for HCC treatment.

## Figures and Tables

**Table I:** Tumor weight and pulmonary metastasis rate in two models of hepatocellular carcinoma.

Model	No. of cases	Tumor weight (g) (mean ± SD)	Metastatic rate, % (n/total)	No. of metastatic foci (mean ± SD)	No. of metastatic cells (mean ± SD)
MHCC97-H	26	1.83±0.75	69.2 (18/26)	4.72±5.50	119.11±185.92
Xenograft	17	2.89±0.84^a^	64.7 (11/17)	2.27±1.01	85.18±79.96^b^

SD, standard deviation;

a P<0.01, compared with the MHCC97-H group;

b P=0.08, compared with MHCC97-H group.

**Table II:** I*n vivo* expression of TGF-β1/Smad mRNA in two mice models.

			95% CI		
mRNA	Models	2^−ΔΔCt^ (mean ± SD)	Lower bound	Higher bound	P-value
TGF-β1	MHCC97	1.92±1.70	1.23	2.61	0.035
	Xenograft	0.97±0.73	0.59	1.35	
Smad2	MHCC97	1.17±1.09	0.73	1.61	0.89
	Xenograft	1.13±0.31	0.98	1.29	
Smad7	MHCC97	1.46±0.97	1.07	1.85	0.17
	Xenograft	1.10±0.52	0.83	1.37	

Student’s t-test was used to assess the statistical significance of differences between two groups. TGF-β1, transforming growth factor-β1; 95% CI, 95% confidence interval for mean; SD, standard deviation.

**Table III.** Comparison of TGF-β1/Smad mRNA between metastatic and non-metastatic samples.

				95% CI		
mRNA	Metastasis	No.	2^−ΔΔCt^ (mean ± SD)	Lower bound	Higher bound	P-value
TGF-β1	None	14	1.10±0.83	0.61	1.58	0.024
	Lower	14	1.16±0.63	0.79	1.53	
	Higher	15	2.32±2.11	1.16	3.49	
Smad2	None	14	1.23±1.35	0.45	2.01	0.65
	Lower	14	0.97±0.55	0.65	1.30	
	Higher	15	1.25±0.47	0.99	1.52	
Smad7	None	14	1.28±1.95	0.59	1.97	0.89
	Lower	14	1.26±0.62	0.91	1.62	
	Higher	15	1.40±0.62	1.06	1.75	

Student’s t-test was used to assess the statistical significance of differences between the two groups. TGF-β1, transforming growth factor-β1; 95% CI, 95% confidence interval for the mean; SD, standard deviation.
